# Skin Mycobiome of Psoriasis Patients is Retained during Treatment with TNF and IL-17 Inhibitors

**DOI:** 10.3390/ijms21113892

**Published:** 2020-05-29

**Authors:** Yuta Koike, Sayaka Kuwatsuka, Katsutaro Nishimoto, Daisuke Motooka, Hiroyuki Murota

**Affiliations:** 1 Department of Dermatology, Nagasaki University Graduate School of Biomedical Sciences, Nagasaki 852-8523, Japan; s-kuwa22@nagasaki-u.ac.jp (S.K.); h-murota@nagasaki-u.ac.jp (H.M.); 2 Department of Dermatology, Nagasaki Ekiasaikai Hospital, Nagasaki 850-0034, Japan; kjwest@ekisaikai-nagasaki.jp; 3 Department of Infection Metagenomics, Genome Information Research Center, Research Institute for Microbial Disease, Osaka University, Suita 565-0871, Japan; daisukem@gen-info.osaka-u.ac.jp

**Keywords:** psoriasis, biologics, TNF inhibitor, IL-17 inhibitor, fungal microbiome, mycobiome

## Abstract

Background: Biological treatment relieves refractory skin lesions in patients with psoriasis; however, changes in the fungal microbiome (the mycobiome) on the skin are unclear. Methods: The skin mycobiome of psoriasis patients treated with TNF inhibitors (TNFi, *n* = 5) and IL-17 inhibitors (IL-17i, *n* = 7) was compared with that of patients not receiving systemic therapy (*n* = 7). Skin swab samples were collected from non-lesional post-auricular areas. Fungal DNA was sequenced by ITS1 metagenomic analysis and taxonomic classification was performed. Results: An average of 37543 reads/sample were analyzed and fungi belonging to 31 genera were detected. The genus *Malassezia* accounted for >90% of reads in 7/7 samples from the no-therapy group, 4/5 from the TNFi group, and 5/7 from the IL-17i group. Biodiversity was low in those three groups. Few members of the genus *trichophyton* were detected; the genus *Candida* was not detected at all. Among the *Malassezia* species, *M. restricta* was the major species in 6/7 samples from the no-therapy group, 4/5 from the TNFi group, and 5/7 from the IL-17i group whose the other largest species revealed *M. globosa*. Conclusions: The mycobiome is retained on post-auricular skin during systemic treatment with TNF and IL-17 inhibitors.

## 1. Introduction

Psoriasis is a common, heterogeneous, and chronic inflammatory skin disease that affects about 2% of the world’s population [1]. It is characterized by thickened, red, scaly plaques on the skin. Psoriasis is also associated with comorbidities such as joint inflammation, cardiovascular disease, and metabolic syndrome. Although the causes of psoriasis are unclear, mouse models and biological treatment of humans show that underlying immunological mechanisms are dependent on the tumor necrosis factor (TNF)/interleukin (IL)-23/IL-17 axis [2]. When using biological treatments for psoriasis patients, clinicians should be aware of bacterial, fungal, and viral infections [3,4,5]. Anti-TNF-α antibodies are first-generation biologics that have a strong therapeutic effect on psoriasis and psoriatic arthritis; however, patients sometimes discontinue therapy due to infectious disease. As second-generation biologics, anti-IL-17 and anti-IL-23 antibodies have led to marked improvements in the skin of psoriasis patients. These antibodies result in a lower risk of infection than anti-TNF-α antibodies [6]. However, several clinical studies show that psoriasis patients treated with anti-IL-17 antibodies get more mucous Candida infections [7,8,9] because IL-17 plays an important role in innate and adaptive responses against Candida; this was observed in individuals with genetic deficiencies that impair IL-17-related immune responses (e.g., chronic mucocutaneous candidiasis) [10].

The skin is inhabited by a diverse community of microorganisms, including bacteria, fungi, and viruses, which maintain human health by producing antibacterial peptides, formation of biofilms, and inhibiting invasion by pathogens [11]. The skin microbiome is a complex ecosystem whereby the physiochemical conditions imposed by the resident microbiome interact with host biology [12]. Recent psoriasis research demonstrates that an aberrant immune response to antimicrobial peptide LL-37 is likely involved in immunopathogenesis of psoriasis [13]. Likewise, it is increasingly evident that keratinocytes, which are exposed directly to and sense the skin microbiome, trigger innate and adaptive immune responses [14]. Among the above-mentioned microorganisms, the bacterial microbiome on the skin has been studied actively in psoriasis patients. Conventional culture-dependent studies suggest that several microorganisms are associated with disease exacerbation, including *Staphylococcus aureus* [15] and *Streptococcus pyogenes* [16]. In recent years, 16S ribosomal RNA sequencing and next-generation 16s or whole-genome metagenomic sequencing has enabled evaluation of the skin microbiome of psoriasis patients, which was undetectable using culture methods [17,18,19,20,21,22], while these results were not consistent. Understanding the skin fungal microbiome, also called the mycobiome, is also important; however, little is known about the community and dynamics of the skin mycobiome in psoriasis patients.

It is possible that the skin mycobiome of psoriasis patients treated with biologics, especially IL-17 inhibitors (IL-17i), is altered; this might initiate fungal proliferation and infection. In addition, alteration of the skin mycobiome may exacerbate psoriasis activity via production of antimicrobial peptides and direct stimulation of keratinocytes by fungi. The fungal internal transcribed spacer (ITS) 1 sequence is a taxonomic signature that enables identification at the species level. Here, we used ITS1 sequencing to compare the taxonomic diversity of the mycobiome in post-auricular skin samples from psoriasis patients treated with TNF inhibitors (TNFi) and IL-17i with that in samples from those not treated with systemic therapies.

## 2. Results

### 2.1. Patient Background and Sequences of Fungi Detected in Skin Samples

Swab samples were obtained from the skin in post-auricular areas with no obvious psoriatic lesions. We obtained seven samples from psoriasis patients not undergoing systemic treatment (no-therapy group). We also obtained five samples from patients treated with TNFi (TNFi group) and seven samples from patients treated with IL-17i (IL-17i group). Patient demographics are presented in Table 1. Average current psoriasis area and severity index (PASI) scores were 5.8 (±3.6 S.D.) in the no-therapy group, 1.8 (±2.1) in the TNFi group (reduced from 10.2 (±5.7) before treatment with TNFi), and 0.2 (±0.5) in the IL-17i group (reduced from 27.2 ± 16.6 before treatment with IL-17i). After extracting DNA from each swab sample, fungal ITS1 deep sequencing was conducted. The average number of reads from all samples was 37543 (±18969 S.D.). The average numbers of reads from individual groups are shown in Table 2; these data indicate that a sufficient number of fungal genes was obtained. Thereafter, we examined taxonomic assignment of a fungal community.

### 2.2. Taxonomic Analysis of Fungi (Upper Rank)

**Figure 1:**
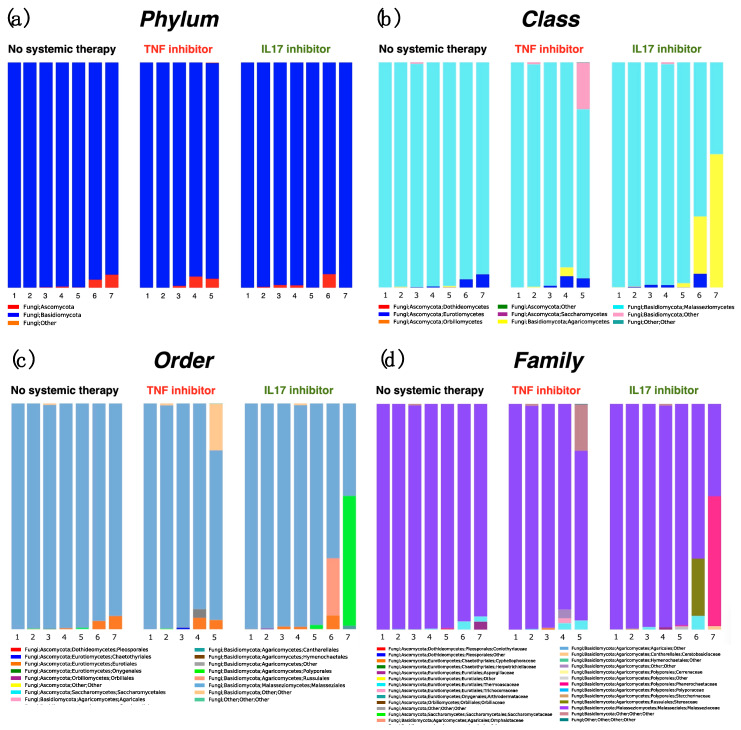
Bar chart showing the relative distribution of fungi at the phylum level (**a**), class level (**b**), order level (**c**), and family level (**d**). Samples were obtained from the post-auricular area of psoriasis patients not receiving systemic therapy (no-therapy group, *n* = 7) and from those treated with TNF inhibitors (TNFi group, *n* = 5) and IL-17 inhibitors (IL-17i group, *n* = 7).

Next, we investigated the taxonomic composition of swab samples according to taxonomic rank: phylum, class, order, family, genus, and species. At the phylum level, all three groups showed equivalent results; almost all sequences belonged to *Basidiomycoma* (Figure 1a). Likewise, *Malasseziomycetes* were most the most common class (Figure 1b). *Malasseziales* were the most common order (Figure 1c) and *Malasseziaceae* were the most common family (Figure 1d). One sample (sample 5) in the TNFi group and two samples (samples 6 and 7) in the IL-17i group showed other high occupancy compositions other than *Malasseziomycetes*, *Malasseziales* and *Malasseziaceae* (Figure 1b–d).

### 2.3. Diversity at the Genus Level

**Figure 2:**
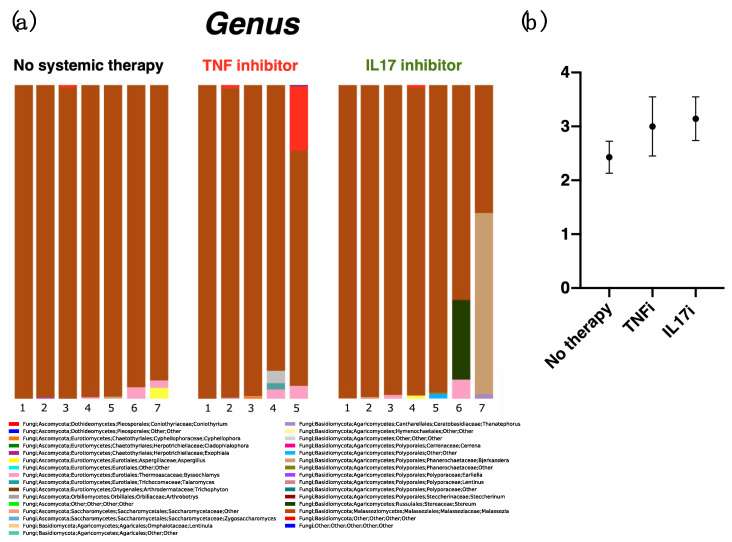
Bar chart showing the relative distribution of fungi at the genus level (**a**). In almost all samples, the major genus was *Malassezia* (brown bar). Other fungi identified in each sample (>20%) included an unidentified fungus belonging to class *Basidiomycota* (red bar) and fungi belonging to the genus *Stereum* (ocher bar) and genus *Bjerkandera* (green bar). Genera present at > 1% included *Byssochlamys* (pink bar); *Aspergillus* (yellow bar); *Talaromyces* (gray bar); *Thanatephorus* (blue bar); and an unidentified fungus belonging to the order *Polyporales* (purple bar). The average number of genera which occupied over 0.1% of individual samples showed no significant difference between the counts for each group: 2.4 (±0.3 S.E.) for the no-therapy group; 3.0 (±0.5) for the TNFi group; and 3.1 (±0.4) for the IL-17i group (**b**).

Next, we analyzed fungi at the genus level. We obtained 31 genera from all samples tested (Figure 2a). The genus *Malassezia* (brown bar) was predominant in all three groups. Other fungi identified in each sample (>20%) included an unidentified fungus belonging to class *Basidiomycota* (20.6%, red bar, in a sample from the TNFi group) and fungi belonging to the genus *Stereum* (57.6%, ocher bar) and genus *Bjerkandera* (25.4%, green bar, in separate samples from the IL-17i group). Genera present at > 1% included *Byssochlamys* (pink bar, in two samples from the no-therapy group (3.7% and 2.3%), two samples from the TNFi group (2.9% and 4.1%), and two samples from the IL-17i group (1.2% and 6.1%)); *Aspergillus* (yellow bar, one sample from the no-therapy group (3.5%) and one sample from the IL-17i group (1.1%)); *Talaromyces* (gray bar, one sample from the TNFi group (2.1%)); and *Thanatephorus* (blue bar, one sample from the IL-17i group (1.6%)). An unidentified fungus belonging to the order *Polyporales* was detected in one sample from the IL-17i group (purple bar, 1.4%). With the exception of genera *Malassezia* and *Aspergillus*, the other detected genera might be adherent microbes encountered during daily life [23]. The reads from the genus *Trichophyton* (a common parasite on humans) were obtained for only one sample in the no-therapy group (0.2%). Intriguingly, no reads were obtained from genus *Candida*. The average number of genera that occupied > 0.1% of any individual sample reveals little diversity, with no significant difference in counts between each group: 2.4 (±0.3 S.E.) for the no-therapy group; 3.0 (±0.5) for the TNFi group; and 3.1 (±0.4) for the IL-17i group (Figure 2b).

### 2.4. Assessment of Malassezia Species

**Figure 3:**
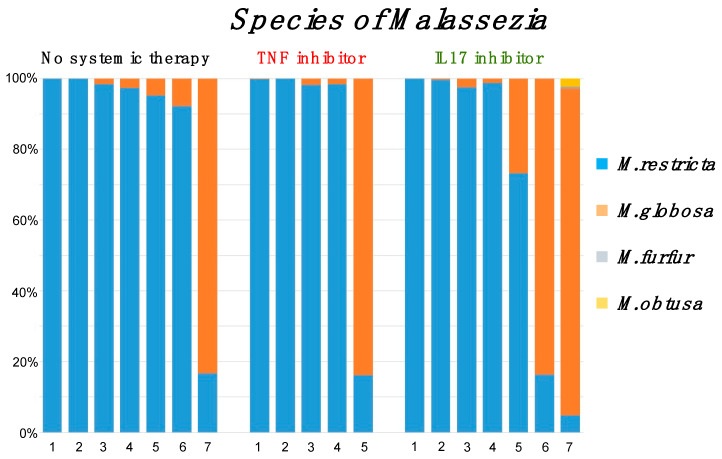
Bar chart showing the relative distribution species within the genus *Malassezia* and description of the color bars. *M. restricta* was dominant in 6/7 samples from the no-therapy group (86%), 4/5 samples from the TNFi group (80%), and 5/7 samples from the IL-17i group (71%). *Malassezia* species other than *M. restricta* showed predominance of *M. globosa.*

As shown above, the genus *Malassezia* were the most common in all swab samples. Therefore, we analyzed the diversity of *Malassezia* species (Figure 3). *Malassezia* species were predominantly *M. restricta* (6/7 samples from the no-therapy group (86%), 4/5 samples from the TNFi group (80%), and 5/7 samples from the IL-17i group (71%)). *Malassezia* species other than *M. restricta* showed predominance of *M. globosa*. Other than those species of *Malassezia* were barely detected in the IL-17i group (*M*. *furfur* 0.005%, *M. obtusa* 0.02%). Thus, biological treatments for psoriasis did not alter the skin mycobiome or the diversity of *Malassezia* species. Meanwhile, all psoriasis patients whose mycobiome was dominated by *M. globosa* were female (male 0%, female 44%, p = 0.03, Fisher’s exact test, Table 3). The dominancy of *M. restricta* or *M. globosa* on the skin appears to be more affected by a patient’s gender than by systemic treatment with anti-TNF antibodies or anti-IL-17 antibodies.

## 3. Discussion

Here, we evaluated the skin mycobiome of psoriasis patients treated with TNFi or IL-17i and compared with that of patients not receiving systemic treatment. Our results revealed that TNFi or IL-17i treatment did not affect the diversity of the mycobiome on the post-auricular skin of psoriasis patients. Almost all extracted fungal DNA belonged to the genus *Malassezia;* other fungi are thought to be acquired via the local environment. Few skin-infecting fungi such as *Trichophyton* and *Aspergillus* were detected, regardless of biological treatment, and no *Candida* was detected at all. Analysis of the *Malassezia* species indicated that distribution was affected more by host gender than by biological therapeutics.

Neither TNFi nor IL-17i altered the mycobiome of non-lesional skin in the post-auricular area of psoriasis patients. A few studies have performed genetic analysis of the skin fungal flora of patients with psoriasis. *Shivaprakash* et al. compared the composition of *Malassezia* species by sequencing the D1/D2 region of 26s rDNA from psoriatic and non-lesional skin from psoriasis patients and healthy people [24]. *Takemoto* et al. conducted 26s rRNA gene sequencing and compared the fungal distribution in psoriatic skin from psoriasis patients with that in skin obtained from healthy individuals [25]. These two studies reported differences in genus *Malassezia* occupancy between these two groups. *Tett* et al. performed a community-wide analysis using high-resolution shotgun metagenomics analysis of samples taken from the olecranon and retroauricular crease; however, they identified poorly characterized clades, including *Malassezia* species [20]. When examining the skin mycobiome of psoriasis patients, interpretation of the results should depend on whether the target skin is from a psoriasis lesion or from a healthy area, and whether the target group is a psoriatic patient or a healthy person. Excessive turnover of epidermal cells and production of a large number of antimicrobial peptides occurs in the lesions of psoriasis patients, which should control aberrant fungal proliferation. Therefore, it might be obvious that the growth environment of healthy microorganisms on psoriatic skin differs greatly from that of healthy skin. For that reason, we examined non-lesional skin from psoriatic patients for comparison; this makes it possible to analyze the direct effects of systemic anti-TNFα antibody and anti-IL-17 antibody treatment on the human skin mycobiome.

We found that the mycobiome on post-auricular skin was unaffected by biologics treatment. There may be several reasons for this. First, it is possible that systemic inhibition of TNF or IL-17 has no effect on the immunological conditions in the normal skin of psoriasis patients. As shown in Table 1, the psoriasis patients in this study derived therapeutic benefit from the biologics, suggesting that the abnormal cutaneous immune system that promotes psoriasis was controlled. The improvement in the psoriatic skin indicated that abnormal activation of the TNF/IL-23/IL-17 axis in the skin returned to normal, and that similar effects would spread to areas of non-lesional skin. The stiffness of the mycobiome elucidated in this study might indicate that the TNF/IL-23/IL-17 axis was not activated in healthy skin, which interacts constantly with the resident fungi. Second, inhibiting TNF or IL-17 may have less effect on the ecosystem of genus *Malassezia* and other skin-resident fungi than on genus *Candida*. The IL-17 pathway regulates antifungal immunity by upregulating proinflammatory cytokines, including IL-6, neutrophil-recruiting chemokines, and antimicrobial peptides, all of which act to control fungal overgrowth. [26]; however, studies of the effect of IL-17 on fungal infection were performed mainly on Candida species (especially *C. albicans*) [27]. A recent study reported that genus *Malassezia* was controlled by IL-23-mediated production of IL-17, a system operating in healthy skin [28]. A third hypothesis is the association with the sample collection site. Here, the specimen collection site was the post-auricular surface; this was chosen to obtain a sufficiently large fungal sample [29]. The skin surface is quite diverse, comprising different microenvironments with distinct pH, temperature, moisture, sebum content, and topography [12]. These niche-specific physiologic differences affect the resident fungi; oily surfaces such as the seborrheic site of the face support fungi that prefer lipids, whereas dry sites like the forearm have a low biomass [11]. The skin mycobiome in humans is occupied widely by genus *Malassezia*, although the diversity varies from site to site [23]. If the collection site comprised an area harboring a more diverse combination of fungi, such as the cubital fossa or foot, the mycobiome might have been affected by systemic treatment with TNFi or IL-17i; however, collection from these sites would be problematic due to fewer extracted fungal genes and/or fungal transmission from the surrounding environment, clothes, and shoes.

We found that all psoriasis patients whose mycobiome was dominated by *M. globosa* were female, with a significant difference by gender, and the others were dominated by *M. restricta*. Gender might influence the abundance of *Malassezia* species, in that female subjects had a reduced amount of *Malassezia*, although changes in *Malassezia* species such as *M. restricta* or *M. globosa* were not documented [29]. *Sugita* et al. suggested that differences between males and females could depend on the prevalence of use of cosmetics, which contain compounds that may inhibit cutaneous fungal growth [30]; however, our sample site is the post-auricular area where cosmetics are not usually used. Male psoriasis patients, most of whom should be associated with metabolic syndrome, might have more seborrheic skin than healthy people, leading their mycobiome to be *M. restricta*-rich, which is the pathogen of seborrheic dermatitis.

The present study found that TNFi and IL-17i did not alter the skin mycobiome in the post-auricular area of psoriasis patients. This study might be the first to evaluate the alternation of skin mycobiome in psoriasis patients with biological therapies. Of course, there are some limitations in this assessment. The number of both samples and subjects was too small to achieve subgroup analysis with respect to treatment, body site, gender and skin condition (lesion or non-lesion). There remains a possibility that these limitations might affect the consequences of the results obtained in this assessment. Despite the limitations, information derived from our assessment will be informative for all clinicians who see patients at dermatology clinics. Moreover, this preliminary data will provide a novel point of view for daily clinical practice. We hope that this study will contribute to the development of skin mycobiome research on psoriasis patients in the future.

## 4. Materials and Methods

### 4.1. Study Subjects

Nineteen patients with plaque-type psoriasis were enrolled in the study. All patients visited Nagasaki University Hospital. Inclusion criteria were as follows: PASI > 10 at any visit; age 16 years. Subjects in the no-therapy group did not receive biologics, etretinate, cyclosporine, or apremilast within the 24 weeks prior to the study. Patients in the TNFi or IL-17i groups had been receiving treatment for > 12 weeks. All subjects provided oral informed consent under protocols approved (approve date; 16 October 2018) by the ethics committee of Nagasaki University Hospital (18101515-2). Sex, age, onset of age, PASI before biologic treatment, and current PASI were recorded.

### 4.2. Sample Collection

Skin samples were taken from each patient by swabbing the post-auricular area 50 times or more with a swab (BD BBD culture swab plus, BD, Tokyo, Japan), from June to July. Since colonization of the skin by fungi is dependent on local psoriatic conditions [24,25], all samples were taken after confirming that the post-auricular skin had no obvious psoriatic lesions. Swab samples were stored at −80 °C until required.

### 4.3. Fungal ITS1 Deep Sequencing, Bioinformatic Analysis, and Taxonomic Assignment

Fungal ITS1 deep sequencing, bioinformatic analysis, and taxonomic assignment were carried out as reported previously [31]. Briefly, DNA was extracted from swab samples using a DNeasy PowerSoil Kit (QIAGEN, Hilden, Germany). Each library was prepared using a two-step PCR method with primer set ITS1-F: 5′-CTTGGTCATTTAGAGGAAGTAA-3′ and ITS2: 5′-GCTGCGTTCTTCATCGATGC-3′ (targeting the fungal ITS1 region), and Nextera XT Index Kit v2 (Illumina, San Diego, CA, USA). Next, 301 bp paired-end sequencing of the amplicon was performed on a MiSeq apparatus (Illumina San Diego, CA, USA). The obtained paired-end sequences were merged using PEAR [32]. These sequences were then clustered into operational taxonomic units (OTU), defined using a 95% similarity cut-off using UCLUST version 1.2.22q [33]. Sequences representative of each OTU were classified taxonomically using RDP Classifier version 2.2 [34] and the ntF-ITS1 database [31]. The bioinformatics pipeline QIIME, version 1.9.1 [35], was used as the informatics environment to calculate relative fungal abundance.

## Figures and Tables

**Table 1:** Patient characteristics.

Group	Male /Female	Age ^1^	Onset Age ^1^	PASI Before Treatment ^1^	PASI Current ^1^
No-therapy group (*n* = 7)	5/2	51.6 ± 21.4 (16–82)	35.0 ± 19.2 (12–61)	N/A	5.8 ± 3.6 (0.4–11.3)
TNFi group (*n* = 5)	2/3	50.2 ± 16.2 (33–82)	33.6 ± 12.6 (21–57)	10.2 ± 5.7 (0.4–18.2)	1.8 ± 2.1 (0–5.6)
IL-17i group (*n* = 7)	3/4	56.9 ± 22.8 (16–83)	38.1 ± 20.3 (12–65)	27.2 ± 16.6 (4.3–59.1)	0.2 ± 0.5 (0–1.5)

^1^ average ± S.D (range), PASI: psoriasis area and severity index, N/A: not applicable

**Table 2:** The average number of reads per group.

Group	Average Reads (±S.D.)
No-therapy group (*n* = 7)	34,523 (±17,986)
TNFi group (*n* = 5)	40,301 (±19,250)
IL-17i group (*n* = 7)	38,349 (±22,162)

**Table 3:** Association between *M. restricta* and *M. globosa* and patient gender.

Gender	*M. restricta*	*M. globosa*
Male (*n* = 10)	10	0
Female (*n* = 9)	5	4

## Abbreviations

TNF	Tumor necrosis factor
IL	Interleukin
ITS	Internal transcribed spacer
*M*.	*Malassezia*
PASI	Psoriasis area and severity index
TNFi	Tumor necrosis factor inhibitors
IL-17i	Interleukin-17 inhibitors
OTU	Operational taxonomic units
